# Supplementary material for: Multifunctional Meta-optic Azimuthal Shear Interferometer
Source: Nano Lett. 2025 Apr 23;25(18):7419–25. doi: 10.1021/acs.nanolett.5c00873 (PMC12063176; doi:10.1021/acs.nanolett.5c00873)
Supplement: Supplementary file 1 — nl5c00873_si_001.pdf [file nl5c00873_si_001.pdf]

Supporting Information for: Multifunctional meta-optic azimuthal shear interferometer

Linzhi Yu,[†] Sergei Shevtsov,[‡] Haobijam Johnson Singh,[†] Peter G. Kazansky,[‡] and Humeyra Caglayan*[,†,¶]

†*Department of Physics, Tampere University, 33720, Tampere, Finland*

‡*Optoelectronics Research Centre, University of Southampton, Southampton SO17 1BJ, UK*

¶*Department of Electrical Engineering and Eindhoven Hendrik Casimir Institute, Eindhoven University of Technology, Eindhoven 5600 MB, The Netherlands*

E-mail: h.caglayan@tue.nl

S1: The theory of photonic spin Hall effect

The principle by which birefringent meta-atoms in the meta-ASI manipulate the incident wavefront can be described using the Jones matrix formalism as follows:

$$\begin{bmatrix} E_{x,\text{out}} \\ E_{y,\text{out}} \end{bmatrix} = \begin{bmatrix} \cos\vartheta & \sin\vartheta \\ -\sin\vartheta & \cos\vartheta \end{bmatrix} \begin{bmatrix} e^{i\varphi_x} & 0 \\ 0 & e^{i\varphi_y} \end{bmatrix} \begin{bmatrix} \cos\vartheta & -\sin\vartheta \\ \sin\vartheta & \cos\vartheta \end{bmatrix} \begin{bmatrix} E_{x,\text{in}} \\ E_{y,\text{in}} \end{bmatrix}, \tag{S1}$$

where $E_{x,\text{in}}$ and $E_{y,\text{in}}$ represent the x- and y-polarized complex amplitudes of the incident light field, while $E_{x,\text{out}}$ and $E_{y,\text{out}}$ are the corresponding components of the transmitted field. The phase delays for x- and y-polarized light are denoted by φ_x and φ_y, respectively, with ϑ representing the meta-atom's rotation angle (shown in Figure 1b, Main Text). For a half-waveplate, $\varphi_y - \varphi_x = \pi$. Assuming the incident light is linearly polarized (LP) along the x-axis, the input field can be expressed as a superposition of right-circularly polarized (RCP) and left-circularly polarized (LCP) components:

$$\begin{bmatrix} E_{x,\text{in}} \\ E_{y,\text{in}} \end{bmatrix} = \begin{bmatrix} 1 \\ 0 \end{bmatrix} = \frac{1}{2} \begin{bmatrix} 1 \\ i \end{bmatrix} + \frac{1}{2} \begin{bmatrix} 1 \\ -i \end{bmatrix}. \tag{S2}$$

The transmitted field after passing through the meta-atom is given by:

$$\begin{bmatrix} E_{x,\text{out}} \\ E_{y,\text{out}} \end{bmatrix} = e^{i\varphi_x} \begin{bmatrix} \cos^2(\vartheta) - \sin^2(\vartheta) \\ -2\cos(\vartheta)\sin(\vartheta) \end{bmatrix}. \tag{S3}$$

To determine the RCP and LCP components of the transmitted field, we apply the circular polarization transformation:

$$\begin{bmatrix} E_{\text{RCP,out}} \\ E_{\text{LCP,out}} \end{bmatrix} = \frac{1}{\sqrt{2}} \begin{bmatrix} 1 & -i \\ 1 & i \end{bmatrix} \begin{bmatrix} E_{x,\text{out}} \\ E_{y,\text{out}} \end{bmatrix} = \frac{1}{\sqrt{2}} \begin{bmatrix} e^{i(\varphi_x + 2\vartheta)} \\ e^{i(\varphi_x - 2\vartheta)} \end{bmatrix}. \tag{S4}$$

Thus, the RCP and LCP components of the transmitted field acquire opposite phase delays

for incident LP light. This phenomenon, known as the photonic spin Hall effect,[1,2] has been widely utilized in metasurfaces and metalenses.[3,4]

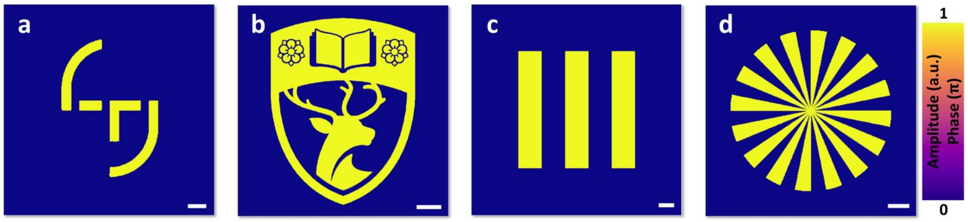

Figure S1: **Patterns used for all-optical image edge detection testing.** (a) Tampere University logo pattern. (b) University of Southampton logo pattern. (c) Resolution line pair pattern. (d) Spoke target pattern. For amplitude images, the patterned regions are transmissive, while the surrounding areas are opaque. For phase images, the patterned regions exhibit a relative phase delay (π in simulations). Scale bars: 200 µm.

S2: Performance analysis of meta-ASIs with varying angular shear amounts

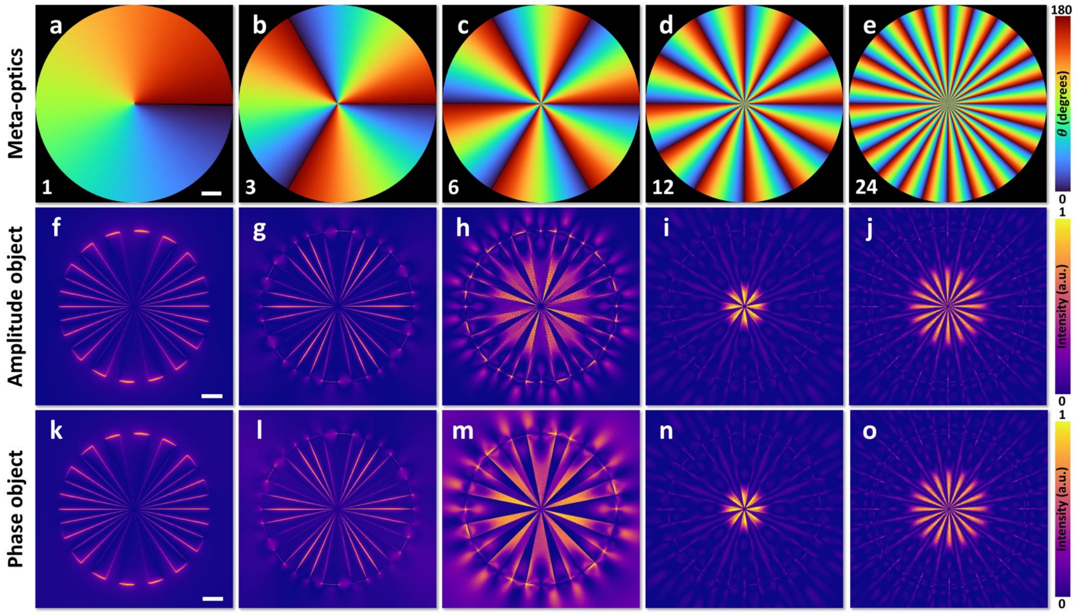

Figure S2: **Performance analysis of meta-ASIs with varying angular shear amounts.** (a–e) Short-axis orientation distributions of meta-optics with different angular shear amounts. (f–j) Simulated edge-detection results for an amplitude image, corresponding to the meta-optics in (a–e). (k–o) Simulated edge-detection results for a phase image using the meta-optics in (a–e). The spoke patterns for the amplitude and phase images are shown in Figure S1d. Scale bars in (a-e): 1 mm. Scale bars in (f-o): 200 µm.

In conventional azimuthal shear interferometers, the wavefront is sheared by a constant angular amount.[5,6] The intensity distribution of the resulting interference pattern is given by:

$$I(r,\theta) \propto |E(r,\theta-\Delta\theta)+E(r,\theta+\Delta\theta)|^2 \tag{S5}$$

where $\Delta\theta$ represents the angular shear amount. The optical transfer function of a conventional azimuthal shear interferometer can be equivalently implemented using meta-optics, as shown in Figures S2a-e, and is described by:

$$\phi'(r,\theta) = \theta C', \tag{S6}$$

where C' is a constant that determines the shear amount. The corresponding shear displace-

ment along the azimuthal direction is:

$$\Delta s' = \lambda f \frac{1}{r} \frac{\partial \phi'}{\partial \theta} = \frac{\lambda f C'}{r}. \tag{S7}$$

This equation indicates that the shear amount is inversely proportional to the radial position r. To analyze the performance of the meta-ASI under varying angular shear amounts, image edge detection was examined for C' values of 1, 3, 6, 12, and 24. Figures S2a-e illustrate the short-axis orientation distributions of the meta-optics corresponding to each C' value. The simulated edge detection results for an amplitude image with a spoke pattern (Figure S1d) are shown in Figures S2f-j, while the results for a phase image with the same spoke pattern are presented in Figures S2k-o. The results clearly demonstrate that the nonuniform shear amount along the radial direction introduces background intensity artifacts, which become more pronounced as the shear amount increases. These artifacts significantly degrade the system's edge detection performance, highlighting the limitations of the constant angular shear approach.

S3: Performance analysis of meta-ASIs with varying azimuthal section numbers

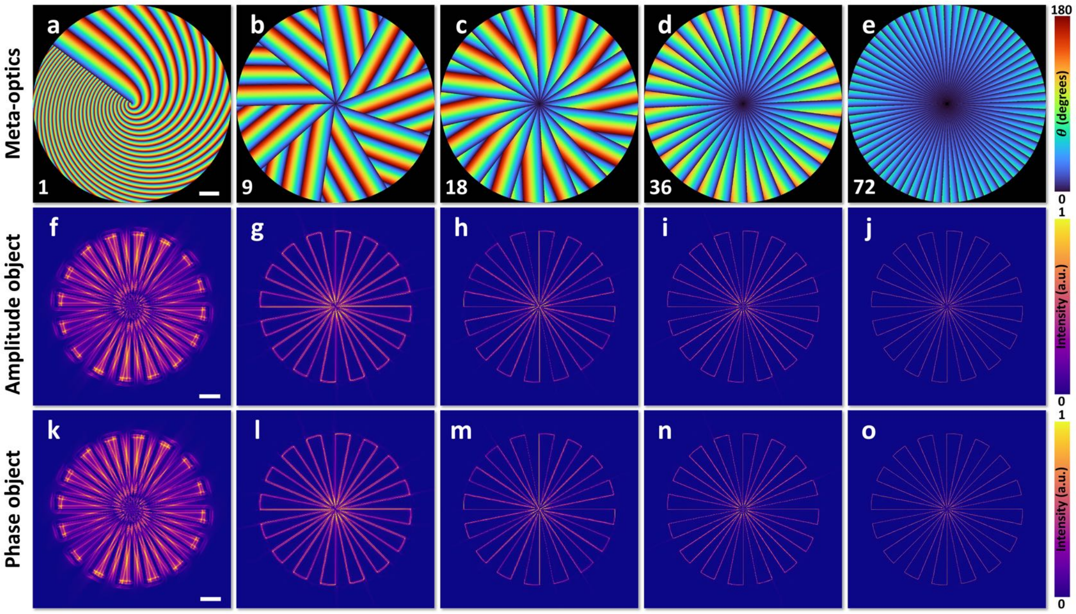

Figure S3: **Performance analysis of meta-ASIs with azimuthal sections ranging from 1 to 72.** (a–e) Short-axis orientation distributions of meta-optics with varying sector numbers. (f–j) Simulated edge-detection results for an amplitude image, corresponding to the meta-optics in (a–e). (k–o) Simulated edge-detection results for a phase image using the meta-optics in (a–e). The spoke patterns for the amplitude and phase images are shown in Figure S1d. Scale bars in (a-e): 1 mm. Scale bars in (f-o): 200 µm.

To achieve uniform azimuthal shear, the phase distribution of the meta-optics is designed as:

$$\phi(r, \theta) = r\theta C, \tag{S8}$$

However, this design introduces a radial displacement error Δr, which is proportional to θ:

$$\Delta r = \lambda f \frac{\partial \phi}{\partial r} = \lambda f \theta C. \tag{S9}$$

This error results in a spiral-shaped distortion in the light field under azimuthal shear interference, as illustrated in Figure S3a,f,k. To mitigate this error, the meta-optics is divided into N azimuthal sections. The influence of the section number on shear interference performance was analyzed using all-optical image edge detection as a test case. The constant C

controlling the shear amount was set to $5.9 \times 10^3\,\mathrm{m}^{-1}$. Five configurations were examined: without azimuthal sectioning ($N = 1$) and with section numbers of $N = 9$, $N = 18$, $N = 36$ (as used in the Main Text), and $N = 72$. The short-axis orientation distributions of the meta-optics for these cases are shown in Figures S3a-e. The corresponding simulated edge detection results for an amplitude image with a spoke pattern (Figure S1d) are presented in Figures S3f-j, while results for a phase image with the same spoke pattern are shown in Figures S3k-o. The results demonstrate that increasing the number of azimuthal sections significantly reduces the radial displacement error. This improvement enhances the sharpness and resolution of the detected edges, illustrating the importance of optimizing the section number in meta-ASI design.

S4: Comparative analysis of edge detection in small structures

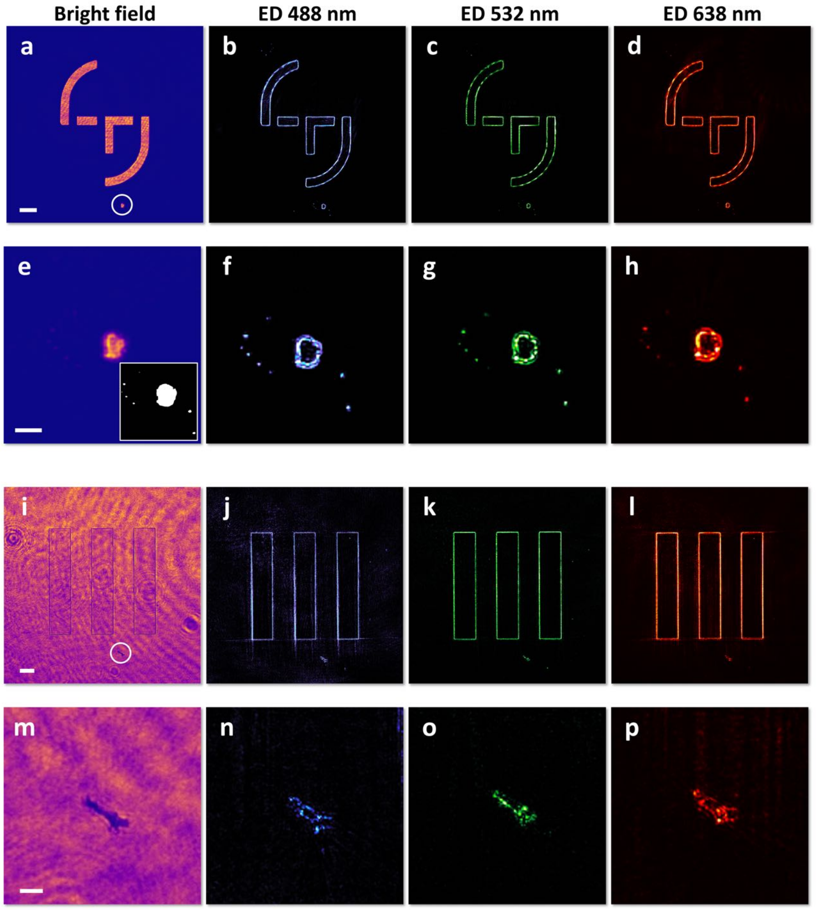

Figure S4: **Comparative analysis of edge detection in fine structures.** (a–d) Contrast-enhanced images of the TAU logo amplitude object. (i–l) Contrast-enhanced images of the resolution line pairs phase object. Fabrication-related features are marked in white. (e–h, m–p) Magnified views of detected features, demonstrating the stability of structural details in the detected edges. The inset in (e) shows a binarized version of a detected structure for improved visibility. Scale bar: 200 µm in full images (a–d, i–l); 50 µm in magnified images (e–h, m–p).

To further assess the performance of edge detection, we compare the detection of fine structures, including fabrication-induced features and surface irregularities, in amplitude and phase objects. Figure S4 presents a comparative analysis of contrast-enhanced versions of the TAU logo amplitude image (Figure 2(d,g,j,m) in the main text) and the resolution line pairs phase image (Figure 3(l,i,m,o) in the main text). Figure S4(a–d) and Figure S4(i–l) show the contrast-enhanced amplitude and phase object images, respectively, where fabrication-

related features are marked in white. Additionally, magnified views of these structures are provided in Figure S4(e–h) and Figure S4(m–p), demonstrating the consistency of the detected structural features. The inset in Figure S4(e) further presents a binarized version of one of the detected features for improved visibility. This comparison highlights the capability of the proposed meta-optic system in reliably capturing fine structural details in amplitude and phase objects.

S5: Numerical simulation method

The theoretical intensity distributions for edge detection (Figure 2a-c, Figure 3a-c, Main Text) and azimuthal shear interference (Figure 5d-f, Main Text) were simulated using Fourier optics.[7] First, the amplitude transfer functions (ATFs) of the meta-ASI for RCP and LCP components were calculated. These ATFs were then multiplied by the spatial spectrum of the input light field to obtain the spatial spectrum of the output RCP and LCP fields. The final intensity distribution was derived by calculating the absolute square of the interfered fields. The meta-ASI design and all simulations were performed using MATLAB.

S6: Aberrated wavefronts generation

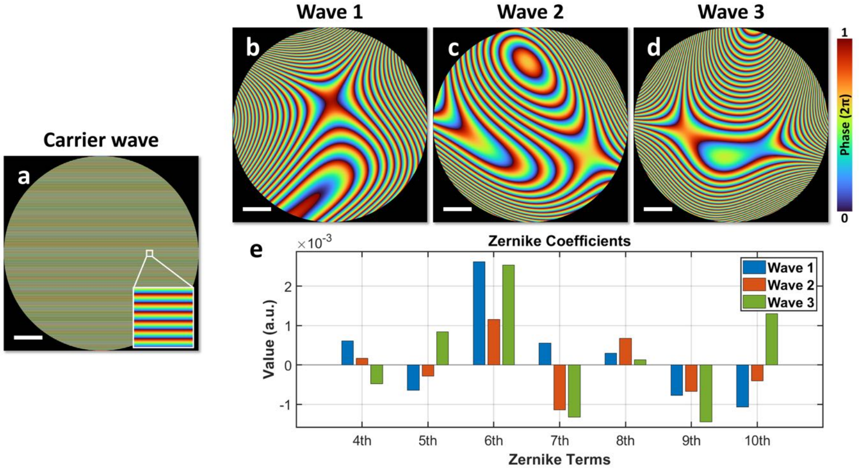

Figure S5: **Aberrated wavefronts used in the experiment.** (a) Phase map of the high-frequency carrier wave. (b-d) Phase maps of wavefronts 1, 2, and 3, respectively. (e) Normalized Zernike polynomial coefficients for the 4th to 10th terms of wavefronts 1, 2, and 3. Scale bars: 1 mm.

The aberrated wavefronts used in the azimuthal shear interference experiment were generated using Zernike polynomials (4th to 10th terms) with randomly assigned coefficients.[8,9] To enhance the interference effect and separate the aberrated wavefronts from unmodulated light, a high-frequency blazed grating was applied, as illustrated in Figure S5a. The phase maps for wavefronts 1, 2, and 3 are shown in Figures S5b,c,d, respectively, with their corresponding coefficients provided in Figure S5e.

S7: Experiment setups

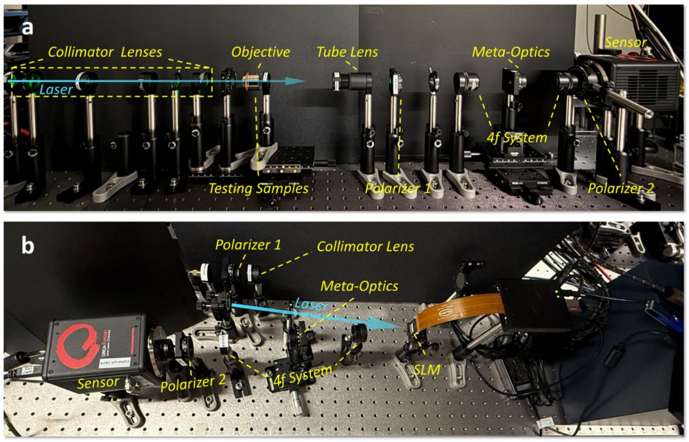

Figure S6: **Experimental setups.** (a) Setup for image edge detection and DIC microscopy. (b) Setup for azimuthal shear interference of aberrated wavefronts.

The performance of the meta-ASI in edge detection and differential interference contrast (DIC) imaging was experimentally evaluated using the setup shown in Figure S6a. Laser beams at wavelengths of 488 nm, 532 nm, and 638 nm were expanded and collimated using a series of achromatic lenses and pinholes to ensure uniformity and sufficient beam size. The 532 nm laser was generated using a Raman laser (WiTec, Oxford Instruments), while the 488 nm and 638 nm lasers were produced by a semiconductor laser (FISBA READYBeam™ Bio1). The collimated beam illuminated the test sample, which was subsequently imaged through an objective (Edge detection: EC EPIPLAN-NEOFLUAR 2.5×/0.06, ZEISS; DIC microscopy of onion epidermal cells: EC EPIPLAN -NEOFLUAR 10×/0.25, ZEISS; DIC microscopy of MDCK cells: EC EPIPLAN-NEOFLUAR 50×/0.8, ZEISS) and an achromatic doublet acting as a tube lens (AC254-150-A, Thorlabs) onto an intermediate image plane. A polarizer placed before the intermediate image plane blocked the horizontal polarization component. The intermediate image was relayed through a 4f system (4f lenses: AC254-075-AB-ML, Thorlabs) to a camera sensor (ORCA-Fusion C14440-20UP, Hamamatsu). The meta-optics was positioned at the Fourier plane using an x-y-z translation stage. A second polarizer, located between the 4f system and the camera, blocked the vertical polarization

component of the modulated wavefront.

Figure S6b illustrates the experimental setup for observing azimuthal shear interference of aberrated wavefronts. A 532 nm laser beam, generated by a Raman laser (WiTec, Oxford Instruments), was passed through a polarizer to align its polarization horizontally. The beam was collimated using a collimator lens (LA1608, Thorlabs) and directed onto a spatial light modulator (SLM, LETO-3, HOLOEYE) using two reflection mirrors. The SLM output was imaged onto a camera sensor (ORCA-Fusion C14440-20UP, Hamamatsu) using a 4f system (4f lenses: LB1676-A, Thorlabs). The meta-optics was positioned at the Fourier plane with the aid of an x-y-z translation stage. A second polarizer, placed between the 4f system and the camera, blocked the vertical polarization component of the modulated wavefront.

S8: Meta-optics fabrication and characterization

The meta-optics used in this study was designed for optimal efficiency at a wavelength of 600 nm. Each meta-atom consists of a nanopore structure embedded within a silica substrate, fabricated using laser direct writing. Specifically, anisotropic nanoporous structures (type X) were imprinted inside the silica glass using a mode-locked Yb:KGW ultrafast laser system (Pharos, Light Conversion Ltd.), operating at 1030 nm with a variable repetition rate ranging from 1 kHz to 1 MHz and a pulse duration adjustable between 190 fs and 10 ps. The laser beam was focused through a 0.16 numerical aperture (NA) aspheric lens into the sample, which was mounted on an x-y-z translation stage (Aerotech Ltd.) for precise position control and scanning speed adjustment. A raster scanning geometry was employed to ensure smooth and uniform modification across the substrate. The slow axis orientation of the type X structures was actively controlled during the writing process by adjusting the polarization of the incident laser beam.[10] This was achieved using a combination of a linear polarizer, an electro-optic modulator, and a quarter-waveplate. The resulting slow axis of the imprinted structures was perpendicular to the polarization of the incident writing light, ensuring consistent birefringence properties. To achieve the required retardance of 300 nm, which corresponds to a half-waveplate at 600 nm, a multilayer structure was implemented. Each individual layer exhibited a retardance of approximately 37.5 ± 1.5 nm, with a layer separation of 60 µm to ensure precise optical modulation.[11–13]

The retardance and slow axis orientation of the fabricated modifications were quantitatively analyzed using an Olympus BX51 optical microscope equipped with a birefringence measurement system (CRi Abrio imaging system), operating at 546 nm. This system enabled precise characterization of the birefringent properties, ensuring that the meta-optics met the required performance criteria.

For scanning electron microscope (SEM) imaging, the laser-processed glass was first polished in the modified region to expose the nanoporous structures. To enhance the visibility of these structures, the sample was etched in a 1 $\mathrm{mol \cdot L^{-1}}$ potassium hydroxide (KOH) solution

for 24 hours. Finally, a thin gold coating was applied to improve conductivity and achieve high-contrast SEM imaging. These steps ensured clear visualization of the metasurface's nanoscale features, further verifying the fabrication quality.

S9: Testing sample fabrication

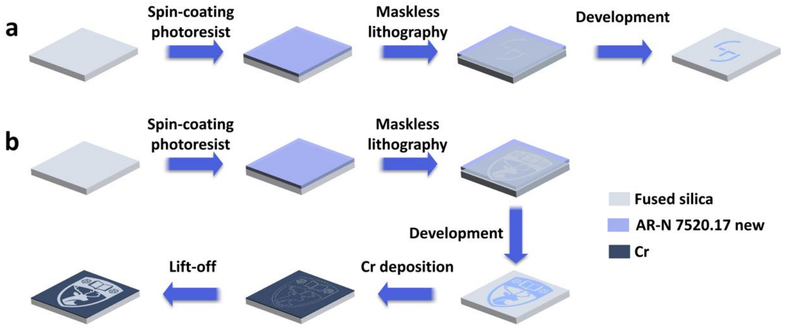

Figure S7: **Fabrication process flows for samples used in the image edge detection experiment.** (a) Process flow for fabricating phase image samples. (b) Process flow for fabricating amplitude image samples.

Figure S7a illustrates the fabrication process flow for the phase sample used in the image edge detection experiment. A layer of photoresist (AR-N 7520.17 new) was spin-coated onto a glass substrate at 4000 rpm, followed by baking at 85°C for 1 minute. The pre-designed pattern was directly written onto the photoresist using a laser direct writing system (Heidelberg Instrument µPG 501). After development with AR 300-47, the photoresist formed a transparent pattern, introducing phase modulation to the transmitted light. A post-bake at 85°C for 1 minute was applied to solidify the pattern. The fabricated phase samples are shown in Figures S8d–f.

Figure S7b depicts the fabrication process flow for the amplitude sample. Two layers of photoresist (AR-N 7520.17 new) were sequentially spin-coated onto a glass substrate at 4000 rpm, with each layer baked at 85°C for 1 minute. The subsequent steps mirrored those of the phase sample. Following development, a 60 nm chromium (Cr) layer was deposited using an electron-beam evaporation system (Instrumentti Mattila IM-9912). A lift-off process with AR 300-73 at 50°C was then performed, transferring the pre-designed pattern into a transparent aperture to modulate the light's amplitude. The fabricated amplitude samples are shown in Figures S8a–c.

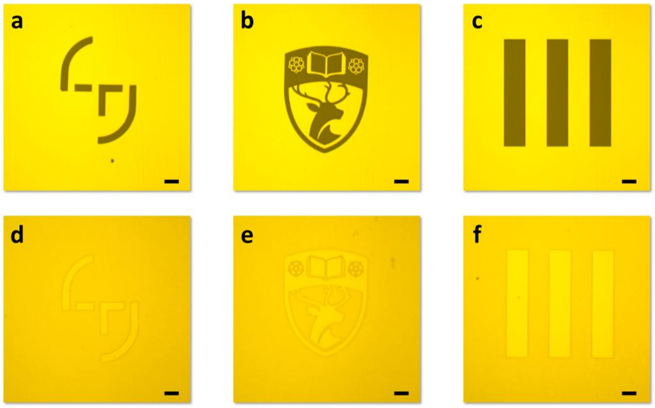

Figure S8: **Samples with patterns used in the image edge detection experiment.** (a-c) Amplitude image samples. (d-f) Phase image samples. Scale bars: 200 µm.

S10: CMOS-integrated meta-optic azimuthal shear interferometer

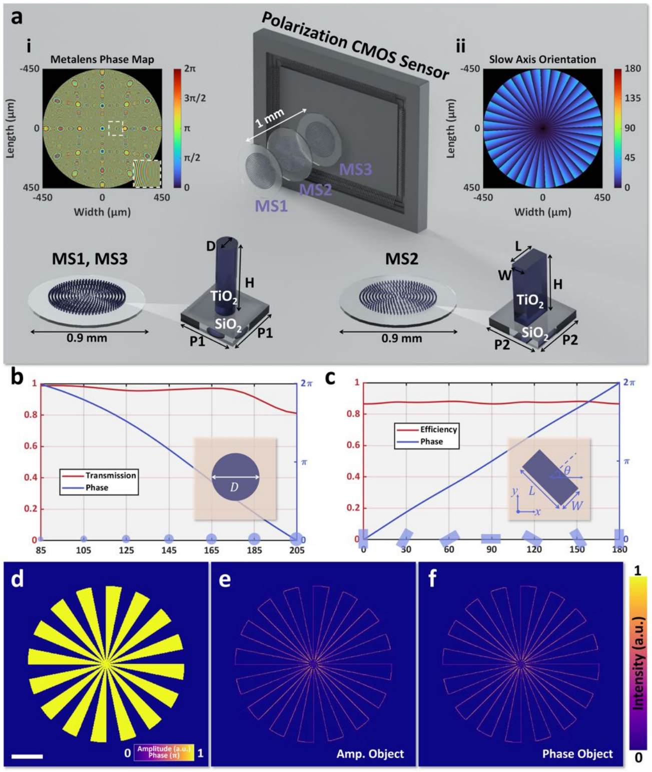

Figure S9: **CMOS-integrated meta-optic azimuthal shear interferometer.** (a) Schematic of the CMOS-integrated meta-ASI, integrating three metasurfaces (MS1, MS2, MS3) onto a polarization-sensitive CMOS sensor. MS1 and MS3 function as metalenses and MS2 acts as the azimuthal shear interferometer. Insets: (i) Phase modulation of MS1 and MS3, and (ii) Short-axis orientation distribution of nanopillars in MS2. (b) Phase response and transmittance of polarization-insensitive cylindrical TiO_2 nanopillars with varying diameters. (c) Phase response and efficiency of TiO_2 nano half-waveplates at different orientations. (d) Spoke pattern amplitude and phase objects used in simulations. (e, f) Simulated edge detection results for amplitude and phase objects, respectively. Scale bar: 50 µm.

To explore the potential for further miniaturization and integration, a compact CMOS-integrated meta-optic azimuthal shear interferometer (meta-ASI) is proposed. This design, which can be realized using electron beam lithography (EBL) techniques, consists of three metasurfaces (MS1, MS2, MS3) directly integrated onto a polarization-sensitive CMOS sensor, as shown in Figure S9. The system is optimized for a 532 nm operating wavelength. MS1 and MS3 function as metalenses with a focal length of 500 µm, forming a miniaturized 4f system, while MS2 serves as the azimuthal shear interferometer. Each metasurface has

a diameter of 0.9 mm, and the total system thickness is less than 2 mm, making it highly compact and suitable for on-chip integration. A thin-film polarizer is incorporated to control incident polarization. The metasurfaces are fabricated using titanium dioxide (TiO_2) nanostructures, ensuring high transmission efficiency. MS1 and MS3 consist of polarization-insensitive cylindrical nanopillars with a height of 600 nm and a periodicity of 225 nm. The diameters of these nanopillars, ranging from 85 nm to 205 nm, are spatially varied to achieve the required phase modulation for lensing, as depicted in Figure S9(a, inset i). The phase response of different nanopillar diameters is presented in Figure S9(b), confirming full-phase modulation capability. MS2, responsible for azimuthal shear interference, comprises rectangular nanopillars functioning as half-waveplates. These structures, with dimensions of 600 nm in height, 235 nm in length, and 115 nm in width, are arranged with a periodicity of 450 nm. Their short-axis orientations are spatially modulated to separate the RCP and LCP components, as illustrated in Figure S9(a, inset ii). The phase response and efficiency of TiO_2 nano half-waveplates with different orientations are presented in Figure S9(c), demonstrating effective polarization control. To validate the feasibility of this CMOS-integrated meta-ASI, numerical simulations were conducted on all-optical edge detection using a spoke-pattern amplitude and phase object, as shown in Figure S9(d). The results, presented in Figure S9(e) and (f), confirm the effectiveness of this compact system in enhancing edge contrast for both amplitude and phase objects. The proposed CMOS-integrated meta-ASI offers significant potential for real-time phase imaging, on-chip wavefront sensing, and compact optical computing. While chromatic aberration due to metalens dispersion remains a challenge, this could be mitigated through advanced achromatic metasurface designs and optimized fabrication processes. Such advancements would further enhance the applicability of this technology in biomedical imaging, endoscopic phase microscopy, and adaptive optics, where system miniaturization and performance optimization are critical.

References

(1) Yin, X.; Ye, Z.; Rho, J.; Wang, Y.; Zhang, X. Photonic spin Hall effect at metasurfaces. *Science* **2013**, *339*, 1405–1407.

(2) Liu, S.; Chen, S.; Wen, S.; Luo, H. Photonic spin Hall effect: fundamentals and emergent applications. *Opto-Electronic Science* **2022**, *1*, 220007–1.

(3) Khorasaninejad, M.; Capasso, F. Metalenses: Versatile multifunctional photonic components. *Science* **2017**, *358*, eaam8100.

(4) Ding, X.; Monticone, F.; Zhang, K.; Zhang, L.; Gao, D.; Burokur, S. N.; De Lustrac, A.; Wu, Q.; Qiu, C.-W.; Alù, A. Ultrathin Pancharatnam–Berry metasurface with maximal cross-polarization efficiency. *Advanced materials* **2015**, *27*, 1195–1200.

(5) Malacara, D. *Optical Shop Testing*; John Wiley & Sons, 2007.

(6) Murty, M.; Hagerott, E. Rotational–shearing interferometry. *Applied Optics* **1966**, *5*, 615–619.

(7) Goodman, J. *Introduction to Fourier Optics*; W. H. Freeman, 2017.

(8) Wang, J.; Silva, D. E. Wave-front interpretation with Zernike polynomials. *Applied Optics* **1980**, *19*, 1510–1518.

(9) Zhao, C.; Burge, J. H. Orthonormal vector polynomials in a unit circle, Part I: basis set derived from gradients of Zernike polynomials. *Optics Express* **2007**, *15*, 18014–18024.

(10) Shimotsuma, Y.; Kazansky, P. G.; Qiu, J.; Hirao, K. Self-organized nanogratings in glass irradiated by ultrashort light pulses. *Physical Review Letters* **2003**, *91*, 247405.

(11) Sakakura, M.; Lei, Y.; Wang, L.; Yu, Y.-H.; Kazansky, P. G. Ultralow-loss geometric phase and polarization shaping by ultrafast laser writing in silica glass. *Light: Science & Applications* **2020**, *9*, 15.

(12) Lei, Y.; Kazansky, P.; Shribak, M. Birefringent elements for optical microscopy by ultrafast laser writing. Conference on Lasers and Electro-Optics/Europe (CLEO/Europe 2023) and European Quantum Electronics Conference (EQEC 2023). 2023; p ch_7_4.

(13) Kazansky, P. G.; Shribak, M. Nanostructured birefringent optical elements and microscopes with nanostructured birefringent optical elements. European Patent EP 4473352 A1, 2024.
